# The Treatment of Clozapine-Withdrawal Delirium with Electroconvulsive Therapy

**DOI:** 10.1155/2017/1783545

**Published:** 2017-11-02

**Authors:** Anish Modak, Arne Åhlin

**Affiliations:** Adult Mental Health Unit, The Canberra Hospital, ACT Health, GPO Box 825, Canberra, ACT 2601, Australia

## Abstract

Clozapine, a commonly used atypical antipsychotic, can precipitate a severe withdrawal syndrome. In this report, we describe a case of delirium with catatonic features emerging after the immediate cessation of clozapine subsequent to concerns of developing neuroleptic malignant syndrome. After multiple treatments were found to be inefficacious, electroconvulsive therapy (ECT) was initiated, resulting in significant improvement. A literature search revealed six previous cases of clozapine-withdrawal syndromes of varied symptomatology treated with ECT. To our knowledge, the present case represents the first reported clozapine-withdrawal delirium treated successfully with ECT.

## 1. Introduction

We present a case of delirium with catatonic features following the abrupt cessation of clozapine treatment, which responded to electroconvulsive therapy (ECT). The complexity and diagnostic difficulty of this case highlight the overlap of symptoms between various syndromes, including delirium, catatonia, and neuroleptic malignant syndrome (NMS), all of which can be associated with clozapine withdrawal. Despite evidence from the literature that delirium following clozapine withdrawal can occur, there have been no published cases of clozapine-withdrawal delirium treated with ECT.

## 2. Case Presentation

Mr. J is a 30-year-old Australian man with schizoaffective disorder, who presented with delirium in the context of abrupt cessation of clozapine due to the development of suspected NMS.

Mr. J initially presented to hospital with an exacerbation of schizoaffective disorder, characterized by formal thought disorder and persecutory delusions. Additionally, Mr. J had mixed mood symptoms; with elements of both mania and depression associated with mood-congruent delusions of grandiosity and guilt, respectively. There had been a deterioration in the community over a period of months, with increasingly disorganised and erratic behavior, poor insight, and a decline in functioning. Mr. J was admitted to an acute psychiatric unit under an involuntary treatment order. He was initially treated with 1.5 g of sodium valproate in divided doses, in combination with 6 mg of risperidone daily, over 5 weeks. Subsequently, he was trialed on a combination of sodium valproate, at the same dose, and 20 mg of olanzapine daily, over 5 weeks. After failing to respond to these medications, Mr. J was commenced on clozapine in week 10 of his admission.

This occurs on the background of one previous admission with psychosis at age 24, with similar symptoms of thought disorder and referential delusions. Mr. J's first episode of mental illness was prolonged, with slow response to risperidone treatment, and complicated by an episode of depression. After a year of recovery requiring residential care in a step-down facility, Mr. J had a period of reasonable interepisodic function, working in a call-centre and living independently, on a treatment regimen of low dose oral risperidone and venlafaxine. He is supported by his family, does not use tobacco, alcohol, or other drugs, and has an unremarkable medical history otherwise. There is a family history of bipolar disorder in his mother.

On week 6 of clozapine initiation, after reaching a dose of 300 mg daily and over a few days, Mr. J developed persistent tachycardia (heart rate up to 130), hypertension (systolic blood pressure up to 168 mmHg), low grade fevers (37.8°C), and rigidity, associated with neutrophilia (up to 9.44*∗*10^9^/L) and a raised creatine kinase (CK) (up to 5870 U/L). At this time, he was prescribed no other medication. Mr. J was treated for a presumptuous diagnosis of NMS with abrupt cessation of clozapine and intravenous fluids on a general medical ward, prior to transfer back to the acute psychiatric unit following resolution of his fevers and autonomic symptoms.

Over the following days, Mr. J deteriorated rapidly in his mental state, with floridly disorganised thoughts (word salad), both increased and decreased psychomotor activity (singing, hugging staff, and statuesque posture), severe insomnia (no sleep for 3 days), visual hallucinations, fluctuating disorientation and inattention, and urinary incontinence. Physical examination revealed negativism, intermittent rigidity, and generalised hyperreflexia, but no clonus, autonomic dysfunction, diaphoresis, or fevers. He required 1 : 1 nursing care and prompting for food and fluid intake. This profoundly delirious, catatonic, and psychotic state failed to respond to oral lorazepam (up-titrated to 2 mg four times a day) or valproate (1.5 g daily in divided doses). Chloral hydrate was trialed for insomnia, with insufficient effect. Additionally, Mr. J received a brief trial of benztropine to treat possible cholinergic rebound symptoms, again, with no apparent benefit. Antipsychotic treatment was withheld due to the concern of NMS. CK levels continued to fluctuate, though slowly resolved over two weeks. A lumbar puncture was performed under sedation, with normal protein and cell count in the cerebrospinal fluid, and no evidence of antineuronal antibodies to suggest autoimmune encephalitis. Other serum investigations, including an ammonia level to exclude valproate-induced hyperammonaemic encephalopathy, were unremarkable. As Mr. J had had a normal magnetic resonance imaging- (MRI-) brain earlier in his admission, cerebral imaging was not repeated.

Electroconvulsive therapy (ECT) was commenced on day 13 after clozapine cessation after obtaining an ECT treatment order. Mr. J's first two ECT treatments were delivered bifrontally in order to minimize cognitive side-effects; subsequently, he received a course of 8 further bitemporal ECT treatments, at a pulse width of 1 ms, with dramatic improvement in his mental state observed from the 4th treatment. His self-care and function improved, and he was able to be managed in a low-dependency environment without 1 : 1 nursing. During this time, lorazepam and valproate treatment were ceased due to their anticonvulsant effects. Mr. J was transferred to a psychiatric rehabilitation facility 4 weeks after his first ECT treatment, with some residual psychotic symptoms, having been initiated on a regimen of lithium and quetiapine.

## 3. Discussion

In the literature, there have only been a few case reports of clozapine-withdrawal syndromes treated with ECT. It is well established that abrupt cessation of clozapine can precipitate a rebound psychosis or mania [1, 2]. However, rarer syndromes have also been associated with clozapine withdrawal, such as delirium [3], catatonia [4], serotonin syndrome [5], NMS [6], and movement disorders such as dystonias and dyskinesias [7]. The diverse presentation of clozapine-withdrawal states is likely secondary to its broad pharmacological profile, with antagonistic effects in serotonergic, cholinergic, dopaminergic, and histaminergic receptors leading to rebound hyperactivity in these systems, along with rebound dysregulation of gamma-aminobutyric-acid- (GABA-) ergic pathways as a result of clozapine's activity on GABA receptors [4, 8, 9]. Treatment of clozapine-withdrawal syndromes should be targeted at particular neurotransmitter systems depending on the symptoms and signs observed; for example, benztropine and cyproheptadine have been suggested as treatments for cholinergic and serotonergic rebound symptoms, respectively [5, 9]. Using an antipsychotic with a high anticholinergic potency, such as chlorpromazine, may reduce the risk of cholinergic delirium when clozapine needs to be stopped abruptly [3, 9].

A recent literature review by Bilbily and colleagues revealed 7 cases of clozapine-withdrawal catatonia reported previously, 2 of which were treated successfully with ECT [4]. For the present review, a literature search was undertaken in April 2017, using PubMed, Medline, and PsycInfo databases using the search terms “Clozapine” AND “Electroconvulsive” AND “Rebound” OR “Withdrawal” OR “Cessation.” An additional 4 further case reports were identified in which clozapine-withdrawal syndromes were treated with ECT. Of the total 6 cases identified, 4 were described as cases of catatonia, 1 was described as a case of mania, and 1 was described as a case of rebound psychosis and NMS, in both males and females aged between 26 and 58 (Table 1). There were no cases of clozapine rebound delirium treated with ECT. One case of clozapine-withdrawal catatonia had no response to ECT; otherwise, the other case reports described partial or full response to treatment. However, it is possible that negative results from trials of ECT in clozapine-withdrawal have not been reported in the literature. Finally, the particular modalities of ECT used were not mentioned in any of the articles (e.g., bitemporal or unilateral).

In the present case, it was clear that a dramatic and profound alteration in mental status was temporally correlated with clozapine withdrawal. Nonetheless, there was a diagnostic conundrum. Mr. J presented with features of delirium (inattention, disorientation, acute onset visual hallucinations, and fluctuating course) and catatonia (negativism, periods of immobility, rigidity, and increased motor activity), as described in the Diagnostic and Statistical Manual of Mental Disorders, 5th edition [14]. However, these syndromes are not necessarily mutually exclusive. Given that both hyperactive and hypoactive delirium share motor features that overlap with catatonia, a catatonic subtype of delirium has been described in the literature [15], alternatively conceptualized as cooccurring delirium and catatonia [16–18], with descriptions of cases treated with memantine and ECT [17, 19]. The presence of suspected NMS in our patient further complicated his diagnosis, given that NMS often presents with features of catatonia and has previously been considered as a subtype of malignant catatonia [20]. Though clozapine-withdrawal delirium has been previously described [3], and delirium of other causes treated with ECT [21], the present case appears to be the first reported clozapine-withdrawal delirium to have been successfully treated with ECT.

## Figures and Tables

**Table 1:** Published case reports of syndromes precipitated by clozapine withdrawal treated with electroconvulsive therapy.

Authors	Psychiatric diagnosis	Age	Sex	Clozapine daily dose (mg)	Reason for clozapine discontinuation	Rebound syndrome	Response to ECT
Bastiampillai et al. [2]	Bipolar Disorder	51	F	15–500	Nonadherence	Recurrent mania	Yes
Cerit et al. [8]	Schizophrenia	46	M	200	Nonadherence	Catatonia	No
Koch et al. [10]	Schizophrenia	33	F	250	Leukopaenia	Catatonia	Partial
Kumar et al. [11]	Schizophrenia	29	M	250	Febrile neutropaenia	Catatonia	Yes
Mendhekar et al. [12]	Schizophrenia	26	M	300	Nonadherence	NMS, psychosis	Yes
Bastiampillai et al. [13]	Schizoaffective disorder	58	F	150	Nonadherence	Catatonic, cholinergic, & serotonergic symptoms	Yes
